# Digital Interventions Addressing Cognitive and Psychological Symptoms in Long COVID: Scoping Review of Multicomponent Approaches

**DOI:** 10.2196/80616

**Published:** 2026-06-08

**Authors:** Sandra León-Herrera, Marta Sánchez-Castro, Ana Luisa Neves, Mª Pilar Rodríguez-Pérez, Vinicius Jobim Fischer, Djenna Hutmacher, Reham Aldakhil, Marina Vaillancourt de Dios, Vinicius Anjos de Almeida, Bárbara Oliván-Blázquez, Rosa Magallón-Botaya, Charles Benoy, Raquel Gómez-Bravo

**Affiliations:** 1 Department of Psychology and Sociology, University of Zaragoza, Zaragoza, Spain; 2 Department of Life Sciences and Medicine, University of Luxembourg, Luxembourg, Luxembourg; 3 Department of Primary Care and Public Health, Imperial College London, South Kensington Campus London, London, SW7 2AZ, United Kingdom, 44 (0)20 7589 5111; 4 RISE-Health, University of Porto, Porto, Portugal; 5 Department of Physiotherapy, Occupational Therapy, Rehabilitation and Physical Medicine, University Rey Juan Carlos, Madrid, Spain; 6 Rehaklinik, Centre Hospitalier Neuro-psychiatrique (CHNP), Ettelbruck, Luxembourg; 7 Faculty of Social and Behavioural Sciences, Utrecht University, Utrecht, The Netherlands; 8 University of Säo Paulo, Säo Paulo, Brazil; 9 Department of Medicine, Faculty of Medicine, University of Zaragoza, Zaragoza, Spain; 10 Universitäre Psychiatrische Kliniken Basel, Basel, Switzerland; 11 Research Group Self-Regulation and Health, Institute for Health and Behaviour, Department of Behavioural and Cognitive Sciences, Faculty of Humanities, Education, and Social Sciences, Luxembourg University, Luxembourg, Luxembourg

**Keywords:** long COVID, postacute COVID-19 syndrome, digital health interventions, cognitive symptoms, psychological symptoms, telehealth, eHealth

## Abstract

**Background:**

Long COVID, or postacute COVID-19 syndrome, presents with persistent cognitive and psychological symptoms such as *brain fog*, anxiety, depression, and fatigue, significantly impacting quality of life and daily functioning. Digital health interventions offer a scalable, accessible solution to bridge care gaps, especially where conventional neuropsychological support is limited. However, evidence regarding their effectiveness for neuropsychiatric symptoms in long COVID remains fragmented.

**Objective:**

This scoping review aimed to systematically identify and map the existing evidence on digital interventions targeting cognitive and psychological symptoms in individuals with long COVID. The review also sought to categorize intervention types, assess reported outcomes, and identify methodological gaps to inform future clinical and research priorities.

**Methods:**

The review followed the Arksey and O’Malley framework and adhered to the PRISMA-ScR (Preferred Reporting Items for Systematic Reviews and Meta-Analyses Extension for Scoping Reviews) guidelines. Comprehensive searches were conducted in 4 databases (PubMed, Scopus, Web of Science, and ScienceDirect) from December 2024 to February 2025. Eligible studies included peer-reviewed and gray literature published in English or Spanish since 2020. Studies were screened and selected based on predefined inclusion and exclusion criteria. Data were extracted using a standardized charting form and synthesized narratively, with thematic grouping by intervention type.

**Results:**

Of 888 records identified, 25 (2.82%) were included. Intervention types encompassed telehealth platforms, mobile health apps, virtual reality, online cognitive and psychological therapies, game-based cognitive training, neuromodulation (transcranial direct current stimulation), and multicomponent programs. Most studies reported improvements in psychological well-being, emotional regulation, and cognitive domains such as attention and memory. However, findings varied, with some interventions showing no significant cognitive gains or sustained effects. Common limitations included small sample sizes, lack of control groups, heterogeneity in outcomes and intervention protocols, and short follow-up durations. The underrepresentation of older adults and underserved populations was also noted.

**Conclusions:**

Digital interventions show promise for addressing cognitive and psychological symptoms in long COVID, particularly when delivered as multicomponent programs. Nonetheless, the evidence base remains preliminary. Future research should prioritize high-quality randomized trials with standardized outcome measures, long-term follow-up, and diverse participant samples. Addressing barriers related to digital literacy and access will be essential to ensure equity and real-world effectiveness.

## Introduction

The COVID-19 pandemic has resulted in an unprecedented global health crisis, with more than 700 million confirmed cases worldwide to date. Beyond the acute phase [1,2], a substantial number of individuals experience persistent symptoms, collectively referred to as *long COVID* or *postacute COVID-19 syndrome* [3,4], which can persist for weeks or months after initial recovery. Among the most reported and debilitating effects are cognitive and psychological impairments [5,6], which significantly impair daily functioning, employment, and overall quality of life.

Cognitive symptoms, colloquially referred to as *brain fog*, include problems with attention, memory, executive functioning, and information processing [7,8]. These manifestations can severely impair daily functioning, work performance, and social participation [9,10]. Concurrently, psychological symptoms such as anxiety, depression, posttraumatic stress, sleep disturbances, and fatigue are highly prevalent in individuals with long COVID, further compounding the burden of disease. These neuropsychiatric sequelae often persist long after the resolution of respiratory symptoms and may occur even in individuals who experienced only mild acute illness [11,12].

Emerging evidence suggests that the mechanisms underlying these symptoms are complex and multifactorial, including neuroinflammation, microvascular damage, immune dysregulation, and psychosocial stressors [13,14]. The unpredictability of symptom progression and the lack of clear diagnostic markers pose challenges for clinical management and rehabilitation planning. Furthermore, many patients report difficulties in accessing appropriate care, particularly neuropsychological and mental health services, due to geographical, logistical, or systemic barriers [15,16].

In this context, digital health interventions have gained considerable attention as innovative and scalable tools to bridge gaps in care. These interventions encompass a wide range of modalities, including mobile health (mHealth) apps, telehealth consultations, online therapy platforms, virtual reality (VR) tools, and other web-based cognitive or behavioral training programs [17]. Digital interventions offer the potential for continuous, remote, and personalized support, which is particularly valuable for populations affected by chronic illness and mobility limitations [18].

Although there is a growing body of research exploring the use of digital health tools in the context of long COVID, most studies to date have addressed general management or physical recovery. Fewer have specifically focused on interventions targeting the cognitive and psychological symptoms that are among the most disabling and persistent sequelae of the condition. As such, health care professionals lack a synthesized and focused body of evidence to inform clinical decision-making in this domain, and researchers face uncertainty regarding which digital approaches show the most promise for addressing neuropsychological aspects of long COVID [17,19,20]. Given the rapid evolution of digital health technologies and the fragmented nature of the current literature, a comprehensive mapping of existing evidence in this area is urgently needed [21]. This is particularly important given the increasing prevalence of long COVID and the growing demand for accessible, nonpharmacological support strategies.

This scoping review aims to address this knowledge gap by systematically identifying, mapping, and synthesizing the existing evidence on digital interventions targeting cognitive and psychological symptoms in individuals with long COVID, while categorizing intervention types, evaluating reported outcomes, and identifying key methodological gaps to inform future research and clinical practice.

## Methods

### Study Design

We conducted a scoping review to examine the evidence on digital interventions addressing cognitive and psychological symptoms in individuals with long COVID. This methodology was chosen due to the exploratory nature of the research question and the heterogeneity of the available evidence in terms of study designs, intervention types, and outcome measures. Scoping reviews are particularly suited to emerging research areas, as they allow a comprehensive overview of the literature and the identification of key knowledge gaps, rather than a narrowly focused assessment of effectiveness.

This review followed the framework proposed by Arksey and O’Malley [22], which comprises 5 key stages: identifying the research question; identifying relevant studies; selecting studies; charting the data; and collating, summarizing, and reporting the results. To enhance transparency and methodological rigor, the review was conducted in accordance with the PRISMA-ScR (Preferred Reporting Items for Systematic Reviews and Meta-Analyses Extension for Scoping Reviews) guidelines [23]. The completed PRISMA-ScR checklist is provided as Checklist 1. Additionally, a publicly accessible study protocol was registered in the Open Science Framework on December 11, 2024 [24].

### Research Questions

To structure the research question, we used the population, intervention, comparison, outcomes, and study design framework [25], summarized in Textbox 1.

Textbox 1. **Population, intervention, comparison, outcomes, and study design (PICOS) criteria for the research question.**

**Population**

- Individuals with long COVID who experience cognitive or psychological symptoms

**Intervention**

- Digital interventions (eg, mobile apps, virtual reality, telehealth, and online cognitive or psychological therapies)

**Comparison**

- No comparison was required for inclusion; comparators, when present, were reported

**Outcomes**

- Cognitive outcomes (eg, attention, memory, and executive function) and psychological outcomes (eg, depression, anxiety, fatigue, and well-being)

**Study design**

- Randomized controlled trials, observational studies, protocols, qualitative studies, and reviews (including scoping, systematic, and narrative reviews), as well as gray literature

The secondary research questions were as follows:

- What digital interventions are available and effective for managing cognitive and psychological symptoms in individuals with long COVID?
- What types of digital interventions are most commonly used?
- What evidence supports their efficacy?
- What methodological limitations and knowledge gaps exist?

### Identifying Relevant Studies

A comprehensive search strategy was designed and peer-reviewed by a research librarian. The following databases were searched between December 2024 and February 2025: PubMed, Scopus, Web of Science, and ScienceDirect. Search terms combined concepts related to long COVID (eg, “Long COVID,” “post-COVID,” “post-acute COVID-19 syndrome,” and “chronic COVID”), digital interventions (eg, “teletherapy,” “digital intervention,” “mobile app,” “telehealth,” “eHealth,” and “virtual reality”), and neuropsychiatric symptoms (eg, “cognitive symptoms,” “*brain fog*,” “anxiety,” “depression,” and “mental health”).

In addition to peer-reviewed records, our search strategy included gray literature available within the databases consulted—particularly ScienceDirect—which indexes materials such as book chapters, conference proceedings, and scientific reports. These records were screened using the same inclusion and exclusion criteria applied to peer-reviewed studies.

The search was limited to studies published in English or Spanish from 2020 onward to capture literature emerging during the COVID-19 pandemic. These languages were selected based on the language competencies of the review team and the high likelihood of relevant literature being published in these languages during the COVID-19 pandemic.

The complete search strings for each database are available in Multimedia Appendix 1 [26,27].

Although hand-searching of reference lists is commonly used as an additional step to identify relevant studies, it was not performed in this review. This decision was based on the comprehensiveness of the database search strategy, which was developed in collaboration with a research librarian and included multiple major databases and gray literature sources. This approach was considered sufficient to capture the relevant body of evidence.

### Study Selection

Study selection was conducted in 2 stages. Initially, 2 reviewers independently assessed titles and abstracts to evaluate their potential relevance. Studies deemed potentially eligible were then examined in full text. Disagreements between reviewers were addressed through discussion based on predefined inclusion criteria (Textbox 2), and a third reviewer was involved when necessary to achieve consensus.

No restrictions were placed on study design or sample size. All identified relevant studies were included to provide a comprehensive overview.

Textbox 2. **Eligibility criteria for the study.**

**Inclusion criteria**

- Original studies (quantitative, qualitative, and mixed methods), reviews (including systematic, scoping, and narrative reviews), or study protocols
- Studies involving digital interventions targeting cognitive and/or psychological symptoms in patients with long COVID, either exclusively or as part of a broader multicomponent approach
- Studies published from 2020 onward in English or Spanish

**Exclusion criteria**

- Studies focusing solely on physical symptoms or nondigital interventions
- Studies not reporting outcomes related to cognitive or psychological health
- Studies published in languages other than English or Spanish

### Data Charting

Data from the selected studies were charted using a standardized data extraction table designed to capture key information related to the research questions. The extracted data included authors, year of publication, study design and setting, population and sample size, type and description of the digital intervention (including duration and frequency), cognitive and/or psychological outcomes assessed, key findings, and reported limitations. The data extraction form was piloted on a subset of 3 studies and refined to improve consistency and capture all relevant variables.

Data were extracted by one reviewer and verified by a second reviewer. Discrepancies were resolved by consensus. The charting process remained iterative, allowing for the inclusion of additional variables as needed.

### Data Synthesis

A narrative synthesis was performed to describe the digital interventions, their outcomes, and methodological characteristics. A descriptive thematic analysis was used to classify interventions into key categories (eg, VR, teletherapy, and mobile apps). Outcomes were synthesized narratively according to their effects on cognitive and psychological domains. The interventions were grouped thematically and compared based on their reported effects on cognitive and psychological symptoms.

Findings are presented in tabular format and complemented by descriptive summaries. Additionally, key gaps in the literature and recommendations for future research were outlined based on patterns identified during synthesis.

Because this is a scoping review, no formal quality assessment or meta-analysis was conducted, in line with best practices for this review type. However, reported limitations and methodological concerns were documented.

### Ethical Considerations

This study involved the review of publicly available literature and did not require ethics approval. No human participants or personal data were involved. This study complies with ethical standards for secondary research and adheres to the principles outlined in the Declaration of Helsinki, where applicable.

## Results

### Sources Selection and Characteristics

The initial database searches yielded 888 records. After removing duplicates, 802 (90.32%) unique records remained for title and abstract screening. Of these, 680 (76.58%) records were excluded based on predefined inclusion and exclusion criteria. In total, 122 (13.74%) full-text records were retrieved and assessed for eligibility, leading to the exclusion of 97 (10.92%) records for reasons such as irrelevance to cognitive or psychological symptoms, lack of a digital intervention, or publication in an unsupported language. Following full-text screening, 25 (2.82%) records were included in this scoping review. These records included peer-reviewed research papers, book chapters, and other relevant literature. Although gray literature (eg, commentaries, editorials, and policy statements) was considered, only the abovementioned records met the inclusion criteria.

**Figure 1.**
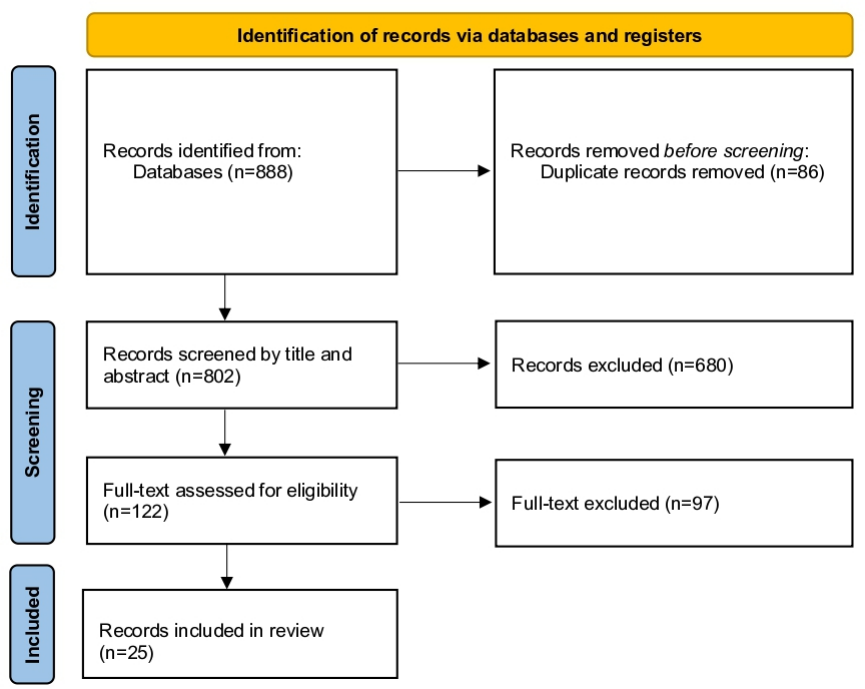
PRISMA (Preferred Reporting Items for Systematic Reviews and Meta-Analyses) flowchart.

The selection process is illustrated in the PRISMA-ScR flow diagram (Figure 1), summarizing the records identified, screened, included, and excluded. In total, 8 (32%) studies were study protocols and had not yet reported outcome data at the time of the review.

To improve readability and facilitate comparison across records, a simplified summary of the included interventions is provided in Table 1. This table groups the records by type of digital intervention, lists the corresponding authors, and highlights the main reported effects on cognitive and psychological symptoms. Detailed methodological characteristics and outcome measures for each study can be found in Multimedia Appendix 2.

**Table 1.** Summary of digital interventions and main reported effects.

Records (author, reference, and year)	Intervention type	Main reported effects
León-Herrera et al [28], 2024; Lai et al [29], 2024; Hatcher et al [30], 2022; Müllenmeister et al [31], 2024; Smith et al [32], 2023; Al-Jabr et al [33], 2022; Ennis et al [34], 2023; and Vanova et al [35], 2024	Telehealth or telerehabilitation	Improvements in quality of life, mental health (anxiety and depression), and self-efficacy, and mixed effects on cognition
Widmann et al [36], 2024; Cano et al [37], 2024; Stavrou et al [38], 2023; Groenveld et al [39], 2022; Prudent et al [40], 2024; and Krotz et al [41], 2023	Virtual reality	Enhanced emotional well-being and physical functioning, inconsistent effects on cognition, and some usability issues
Daniels et al [19], 2024; Mata et al [42], 2023; Smith et al [32], 2023; and Montes-Ibarra et al [43], 2024	Mobile health apps	Gains in attention, mood, and self-reported recovery, often combined with mindfulness or educational components
Tsang and Tabio [44], 2024; Prudent et al [40], 2024; and Al-Jabr et al [33], 2022	Online psychological therapies	Decreased anxiety, posttraumatic stress disorder, and depression and high feasibility and acceptability
Victoria et al [45], 2024	Game-based cognitive training	Improved processing speed and task-switching and no sustained improvement in attention
Eilam-Stock et al [46], 2021	Neuromodulation (transcranial direct current stimulation)	Enhanced cognition, mood, and energy levels in a small-scale pilot study
León-Herrera et al [28], 2024; Smith et al [32], 2023; Daniels et al [19], 2024; Krotz et al [41], 2023; Schröder et al [47], 2023; and Dahmen et al [48], 2022	Multicomponent programs	Broad improvements across mental health, cognitive symptoms, and physical activity

### Population Characteristics

The studies included in this scoping review investigated individuals with persistent post–COVID-19 symptoms across various countries, including Germany, Spain, the United Kingdom, the United States, Canada, Greece, Italy, the Netherlands, and Taiwan. Most included studies were conducted in Europe and North America, indicating a geographic concentration of research and limited evidence from low- and middle-income countries. Populations commonly presented with a mix of cognitive symptoms (eg, *brain fog*, memory impairment, and concentration difficulties) and psychological symptoms (eg, anxiety, depression, fatigue, and sleep disturbances), along with impaired physical function and reduced quality of life.

Sample sizes varied significantly, from as few as 11 participants in a pilot telehealth group therapy study [44] to >600 participants in a blended digital rehabilitation program [32]. Some studies focused on specific age groups—for example, the CICERO trial [35] limited participants to adults aged 30 to 60 years—while others explored experiences across younger and older adults [42]. A common trend was the overrepresentation of women, with some studies noting that digital tool acceptance was higher among women and those with prior digital experience [47]. Although some studies reported participants’ age, gender, and previous digital experience, comprehensive demographic data, such as digital literacy and socioeconomic background, were not systematically reported, limiting subgroup analysis and generalizability.

### Types of Digital Interventions

Digital health interventions varied widely in format, intensity, and content. The most common approaches included telehealth and online rehabilitation platforms, mHealth tools, VR, game-based cognitive therapies, digital psychotherapy, multicomponent programs, and neuromodulation techniques.

Telehealth and online rehabilitation platforms typically combined cognitive training, physical exercise, and mental health support delivered via weekly video calls and digital resources [28,29]. mHealth tools were also frequently implemented; for example, an immersive VR-based app integrating mindfulness and cognitive training was used [37], alongside mobile apps and wearable monitoring tools [19].

VR interventions were explored in several records, showing benefits in physical recovery and emotional well-being [36,38,39], although some users reported side effects such as dizziness and headaches. Game-based cognitive therapy was also represented by a video game designed to enhance cognitive function, which led to improvements in processing speed and executive function.

Digital psychotherapy approaches were also identified, including trauma-focused cognitive behavioral therapy (CBT) delivered online [40] and telehealth group CBT incorporating verified COVID-19 information and peer support [44]. In addition, multicomponent programs combined web-based exercise classes, symptom education, and in-person community support [32]. Finally, neuromodulation techniques, including remotely supervised transcranial direct current stimulation, were reported to improve fatigue and depression in a small case series [46].

The duration and frequency of interventions ranged from 2 weeks to 3 months, with varying intensity and adherence strategies, such as weekly sessions or daily app use.

### Intervention Efficacy

Outcomes evaluated across records spanned several domains. Cognitive function was frequently assessed, including memory, executive function, attention, and processing speed [35,37,45]. Psychological health outcomes were also commonly reported, particularly depression, anxiety, posttraumatic stress disorder, sleep quality, and fatigue [28,40].

In addition, several records examined physical health indicators such as breathlessness, exercise tolerance, sleep quality, and overall physical performance [29,38]. Broader measures of quality of life and functioning—including self-efficacy, daily activities, occupational performance, and health care use—were also assessed [31,32]. Finally, usability and acceptability outcomes, such as satisfaction, adherence, and technological barriers, were reported in a subset of sources [39,47].

Many records reported positive effects across multiple outcome domains. Cognitive improvements were observed in several studies, including enhanced processing speed and attention [45], global cognition [37], and memory performance [28]. However, these effects were not consistent across all studies, with some reporting no significant cognitive gains [38]. Variability in intervention type, duration, and assessment tools likely contributed to these mixed findings.

Psychological benefits included reductions in depression and anxiety [28,40], as well as improvements in emotional well-being. Physical outcomes also improved in several trials, including enhanced sleep quality [29], decreased breathlessness [38], and increased physical activity [32]. Additionally, quality of life and self-efficacy improved, particularly in studies integrating behavioral support and health education [19,28].

Nonetheless, effects were not universally sustained, and several studies reported limited or nonsignificant changes in certain domains, such as sustained attention or long-term mental health outcomes.

### Methodological Limitations and Knowledge Gaps

The methodological quality and design heterogeneity of the included studies present important considerations for interpreting the findings of this review. Although several randomized controlled trials [28,29,45] contributed robust evidence, a substantial proportion of the literature consisted of pilot studies, observational designs, or study protocols without reported outcomes [30,34,48]. This variability limits the overall strength of conclusions and underscores the early, exploratory nature of research in this domain.

Sample sizes were frequently small, particularly in feasibility or case studies [44,46], reducing statistical power and generalizability. Several studies lacked control groups or used nonrandomized designs, making it difficult to establish causal relationships between interventions and outcomes. Blinding—both of participants and researchers—was often absent or not reported, introducing a potential risk of bias.

Additionally, many studies relied heavily on self-reported outcome measures to assess cognitive and psychological symptoms. Although these tools provide valuable patient-centered data, they are subject to recall bias and may not accurately reflect clinical change. The use of diverse instruments across studies also complicates cross-study comparisons. Furthermore, follow-up periods were generally short, limiting insights into the sustainability of treatment effects over time. Overall, although early findings are promising, methodological constraints suggest that current evidence should be interpreted cautiously.

Despite growing interest in digital health interventions for long COVID, important gaps remain that hinder the development of standardized, scalable treatment approaches. One key limitation is the lack of long-term follow-up data. Most studies evaluated outcomes immediately after the intervention or within a few weeks, leaving unanswered questions about the durability of effects, relapse rates, and maintenance strategies.

Intervention protocols were highly heterogeneous in terms of content, delivery mode, duration, and intensity, making it difficult to identify the most effective components or to compare results across studies. The absence of standardized outcome measures further complicates synthesis and meta-analysis efforts. Moreover, although digital interventions are often assumed to be widely accessible, few studies addressed issues related to digital literacy, internet access, or usability challenges—factors particularly relevant for older adults and underserved populations.

Certain groups, including older adults, individuals with severe or complex post–COVID-19 symptoms, and those from lower socioeconomic backgrounds, were underrepresented in most samples. Cultural and gender-specific factors affecting the acceptability and efficacy of interventions were also largely unexplored. Finally, few records examined the mechanisms through which digital interventions exert their effects, leaving open important questions about how improvements in cognitive or emotional functioning are achieved and whether they translate into functional recovery.

Collectively, these gaps highlight the need for well-designed, adequately powered trials with longer follow-up, consistent outcome measures, and inclusive recruitment strategies. Future research should also explore how to personalize digital interventions and integrate them sustainably into health systems to meet the needs of diverse populations affected by long COVID.

## Discussion

### Principal Findings

This scoping review synthesized the current landscape of digital interventions targeting cognitive and psychological symptoms in individuals with long COVID. The findings reflect a field in active development, with diverse intervention types, from telehealth to VR, showing potential benefits across cognitive, emotional, and functional domains [6,49]. Although symptoms such as *brain fog*, memory deficits, fatigue, and anxiety were consistently addressed, intervention efficacy varied widely, underscoring the need for more tailored, evidence-based approaches.

A notable strength of this emerging field is the international scope of research, encompassing studies conducted across Europe, North America, and Asia. Despite differences in study design and health care infrastructure, there was considerable consistency in the reported symptom burden among participants. Cognitive dysfunction and psychological distress, such as anxiety, depression, and fatigue, were prevalent across cohorts. These findings are consistent with prior research that has identified neurological and psychiatric effects as central and disabling features of long COVID [50,51].

Digital interventions were diverse in format, encompassing telehealth platforms, mobile apps, VR, digital cognitive therapies, and online behavioral support programs. Interventions often addressed multiple domains simultaneously, incorporating physical, psychological, and cognitive components. This multimodal approach aligns well with the multisystemic nature of long COVID and reflects a growing emphasis on integrative, patient-centered care [52]. For example, programs like those described by León-Herrera et al [28] and Smith et al [32] combined exercise, cognitive rehabilitation, and education, yielding improvements in quality of life and mental health outcomes.

Despite these promising findings, the effectiveness of digital interventions was not uniform. Although several studies reported improvements in processing speed, attention, emotional well-being, physical activity, and sleep quality, other outcomes—such as sustained attention and long-term psychological recovery—showed inconsistent or nonsignificant effects. These mixed results may reflect the variability in intervention type and intensity, as well as individual differences in symptomatology, baseline functioning, and engagement levels. Previous research has consistently highlighted the heterogeneous nature of long COVID symptoms, with some individuals experiencing more persistent and debilitating effects than others. This variability in symptom severity, along with differences in how patients engage with interventions, may explain the inconsistencies observed across studies. Moreover, the complexity of long COVID, involving multiple systems of the body, suggests that a one-size-fits-all approach to digital interventions may not be equally effective for everyone [53].

The methodological quality of the included records varied widely. Although this scoping review did not conduct a formal risk of bias assessment, a substantial number of included studies used nonrandomized or uncontrolled designs, limiting the strength of causal inferences and contributing to heterogeneity in findings. Although randomized controlled trials provided valuable insights (eg, Victoria et al [45], León-Herrera et al [28], and Lai et al [29]), many studies were limited by small sample sizes, lack of control groups, or reliance on self-reported measures. In particular, the short follow-up periods commonly used across studies limited the ability to assess the durability of observed improvements. Furthermore, the reliance on subjective outcome measures, often without objective neurocognitive or physiological assessments, raises concerns about measurement reliability and the potential for response bias.

In terms of equity and access, several important gaps remain. Many interventions assumed a basic level of digital literacy and reliable access to internet-enabled devices—assumptions that may not hold true for older adults, people with disabilities, or those in socioeconomically disadvantaged settings [54,55]. Notably, long COVID and digital access disparities often intersect with age, socioeconomic status, and geography, especially as digital interventions are intended to bridge, rather than widen, these gaps. Few studies systematically addressed these barriers, and older adults were often underrepresented despite being disproportionately affected by both COVID-19 and digital exclusion. Studies have shown that older adults, individuals from low-income households, and those in rural areas are significantly less likely to have consistent access to internet-enabled devices or the digital literacy needed to engage with these tools. Designing low-bandwidth applications, providing user training, and incorporating hybrid support models may help mitigate these disparities and expand reach [56,57].

Importantly, the mechanisms through which digital interventions exert their effects remain poorly understood. Whether improvements in cognitive or emotional domains result from specific therapeutic content, increased social engagement, improved physical fitness, or a combination of these factors is still unclear. Recent investigations have begun exploring these mechanisms, although definitive conclusions remain elusive [58,59]. Understanding these mechanisms will be essential for refining and targeting future interventions.

Overall, this review reveals a rapidly developing but still nascent field. To build a more robust evidence base, future research should prioritize high-quality randomized trials with adequate sample sizes, long-term follow-up, and standardized outcome measures [60,61]. Equally important is the inclusion of diverse populations and the evaluation of implementation factors such as usability, adherence, and cost-effectiveness. Addressing these gaps will be essential to integrate digital health solutions into routine care pathways and to ensure that they are accessible and effective for all individuals affected by long COVID.

### Strengths and Limitations

This scoping review offers a comprehensive and up-to-date synthesis of digital interventions aimed at addressing long COVID, encompassing a wide range of technological approaches and international contexts. A major strength is the breadth of included study designs, which enables the mapping of both experimental and exploratory initiatives, as well as emerging clinical protocols. By including various forms of digital care—such as telehealth, mobile apps, VR, and neuromodulation—this review captures the evolving landscape of post–COVID-19 rehabilitation and highlights the multidisciplinary nature of digital health innovation.

However, several limitations must be acknowledged. First, the heterogeneity of study designs, populations, and outcome measures limited the ability to perform direct comparisons or draw definitive conclusions about intervention efficacy. Second, as with many scoping reviews, the aim was to map available evidence rather than evaluate quality in depth; thus, a formal risk of bias assessment was not conducted. Finally, the inclusion of ongoing trials and study protocols, while useful for identifying research trends, means that many findings are preliminary and not yet peer-reviewed.

### Clinical and Policy Implications

Physicians and health care systems may consider testing scalable, low-cost digital solutions such as online CBT, symptom tracking apps, or telerehabilitation platforms as complements to conventional care. To ensure successful implementation, it is essential to provide patients with support for uptake, address barriers to access, and align digital interventions with existing service delivery pathways. Integrating these tools can expand the reach of health care and maintain its continuity, especially in underserved populations.

### Future Directions

Future research should build on these early insights by prioritizing well-powered randomized controlled trials that include diverse, representative populations and longer follow-up periods to assess the sustainability of intervention effects. There is a clear need for standardized outcome measures that capture the multidimensional nature of long COVID, including validated tools for cognitive, emotional, and physical health, as well as functional status and quality of life. Research should also explore adaptive and personalized digital interventions that can respond to individual symptom profiles and levels of digital literacy.

In addition, future research should consider mixed methods approaches, as qualitative studies can provide insights into patient experiences and inform the design of well-powered randomized controlled trials. Conversely, trends and patterns observed in large trials can guide research exploring the lived experiences of underrepresented or marginalized populations, including older adults, individuals living in rural or remote areas, and people with disabilities.

Moreover, implementation science approaches are needed to evaluate how digital interventions can be integrated into real-world health care systems, addressing barriers such as technological access, health care professional training, and user engagement. Cost-effectiveness analyses and health equity assessments will be crucial to ensure that digital solutions do not widen existing disparities. Finally, investigating the mechanisms of action—such as whether improvements stem from behavioral change, neuroplasticity, or social support—can help refine digital treatment strategies and optimize outcomes for individuals living with long COVID.

### Conclusions

This scoping review highlights the growing use of digital interventions—such as telehealth, mobile apps, VR, and online therapies—for managing cognitive and psychological symptoms in individuals with long COVID. Although early evidence suggests potential benefits across domains such as mood, attention, and quality of life, findings remain heterogeneous, and sustained improvements are inconsistently reported.

Current evidence is limited by methodological weaknesses, small sample sizes, and short follow-up periods. Additionally, there is a lack of standardized outcome measures and insufficient representation of older and underserved populations.

Future research should prioritize well-powered, methodologically rigorous trials that examine long-term effects, address equity and accessibility challenges, and explore how digital tools can be sustainably integrated into health systems. Personalized, multicomponent digital approaches may offer the most promise for supporting recovery in this complex and heterogeneous condition.

## Supplementary material

10.2196/80616 Multimedia Appendix 1 Complete search strings for each database.

10.2196/80616 Multimedia Appendix 2 Detailed methodological characteristics and outcome measures for each study.

10.2196/80616 Checklist 1 PRISMA-ScR checklist.

## Abbreviations

CBT: cognitive behavioral therapy
mHealth: mobile health
PRISMA-ScR: Preferred Reporting Items for Systematic Reviews and Meta-Analyses Extension for Scoping Reviews
VR: virtual reality
