# Supplementary material for: Digital Interventions Addressing Cognitive and Psychological Symptoms in Long COVID: Scoping Review of Multicomponent Approaches
Source: Interact J Med Res. 2026 Jun 8;15:e80616. doi: 10.2196/80616 (PMC13245839; doi:10.2196/80616)
Supplement: Multimedia Appendix 1 [file ijmr-v15-e80616-s001.docx]

**Appendix 1. Full search strategy.**

| **Database** | **Search Query** |
| --- | --- |
| Web of Science | TS = ("long COVID" OR "post-COVID" OR "post-acute COVID-19 syndrome" OR "chronic COVID") AND TS = ("digital intervention" OR "digital therapy" OR "virtual reality" OR "teletherapy" OR "eHealth" OR "app-based intervention" OR "telehealth" OR "digital health") AND TS = ("cognitive symptoms" OR "psychological symptoms" OR "mental health" OR "brain fog" OR "memory deficits" OR "depression" OR "anxiety") |
| Scopus | TITLE-ABS-KEY("long COVID" OR "post-COVID" OR "post-acute COVID-19 syndrome" OR "chronic COVID") AND TITLE-ABS-KEY("digital intervention" OR "digital therapy" OR "virtual reality" OR "teletherapy" OR "eHealth" OR "app-based intervention" OR "telehealth" OR "digital health") AND TITLE-ABS-KEY("cognitive symptoms" OR "psychological symptoms" OR "mental health" OR "brain fog" OR "memory deficits" OR "depression" OR "anxiety") |
| ScienceDirect | ("long COVID" OR "post-COVID") AND ("digital intervention" OR "digital therapy" OR "telehealth" OR "virtual reality") AND ("cognitive symptoms" OR "psychological symptoms" OR "mental health") |
| PubMed | ("long COVID"[Title/Abstract] OR "post-COVID"[Title/Abstract] OR "post-acute COVID-19 syndrome"[Title/Abstract] OR "chronic COVID"[Title/Abstract]) AND ("digital intervention"[Title/Abstract] OR "digital therapy"[Title/Abstract] OR "virtual reality"[Title/Abstract] OR "teletherapy"[Title/Abstract] OR "eHealth"[Title/Abstract] OR "app-based intervention"[Title/Abstract] OR "telehealth"[Title/Abstract] OR "digital health"[Title/Abstract]) AND ("cognitive symptoms"[Title/Abstract] OR "psychological symptoms"[Title/Abstract] OR "mental health"[Title/Abstract] OR "brain fog"[Title/Abstract] OR "memory deficits"[Title/Abstract] OR "depression"[Title/Abstract] OR "anxiety"[Title/Abstract]) |
