# Supplementary material for: Digital Interventions Addressing Cognitive and Psychological Symptoms in Long COVID: Scoping Review of Multicomponent Approaches
Source: Interact J Med Res. 2026 Jun 8;15:e80616. doi: 10.2196/80616 (PMC13245839; doi:10.2196/80616)
Supplement: Multimedia Appendix 2 [file ijmr-v15-e80616-s002.docx]

**Supplementary Table S1**. **Detailed characteristics of included studies on digital interventions for cognitive and psychological symptoms in Long COVID**

| **Author (year) [Ref]** | **Study setting** | **Study design** | **Population and sample size** | **Type and description of the digital intervention (including frequency and duration)** | **Type of symptom and outcomes measures** | **Key findings** | **Limitations** |
| --- | --- | --- | --- | --- | --- | --- | --- |
| Dahmen et al. (2022) [1] | Bavaria, Germany. | Protocol for a randomized controlled trial. | 132 (66 per group) patients with a positive screening for PACS. | Physical training, psychological interventions, chatbot for symptom management, digital self-help programs. The intervention will occur over several weeks, although the specific number of sessions and exact duration are not detailed in the text. | Symptoms related to PACS; Activities of daily life (ADL); Return to work (RTW); Social participation; Mental health and life satisfaction/quality of life; Motivation, coping strategies, and self-efficacy (mediating factors). | As this is a randomized clinical trial protocol, no results are available yet. | The study does not explicitly mention limitations. |
| Murray et al. (2022) [2] | London, United Kingdom. | Protocol for a mixed-methods single-arm prospective study. | 1000 patients by year 1 with long COVID-19. | Digital health intervention developed by an interdisciplinary team, including patient and public involvement. | Cognitive impairment; Anxiety; Depression; Fatigue; Health-related quality of life; Breathlessness; Cognitive impairment. | As this is a randomized clinical trial protocol, no results are available yet. | Missing data in PROMs.  Clinical data may be incomplete or inconsistent.  Absence of a control group. |
| Widmann et al. (2024) [3] | Bonn, Germany. | Book chapter. | Long COVID-19 patients. | Neuropsychological rehabilitation with telemedicine, virtual reality, and mobile applications. | Cognitive symptoms: disturbances in attention, executive functions, memory, language and visuospatial abilities.  Psychological symptoms: anxiety, depression, sleep disturbances, fatigue, stress and trauma. | Tele-rehabilitation and VR interventions were found to be effective in improving cognitive and psychological symptoms. VR was noted for its benefits in coping with isolation and loneliness.  Mobile apps showed positive impacts on mental health and cognitive outcomes. | Reported limitations involved sample and symptom variability, technological barriers, high costs, and limited research on long-term |
| Mata et al. (2023) [4] | South Eastern United States. | Qualitative study | 38 patients with long COVID-19. | Virtual Integrative Medical Group Visits (IMGV). | Thematic analysis of younger adults’ experiences with long COVID-19 and virtually delivered IMGV. | A total of 21 pre- and 17 post-interviews were conducted. Both younger and older adults found IMGV supportive, validating, and convenient, with no significant differences between age groups. Interviews highlighted the need to address long COVID-19 in older adults due to potential symptom overlap with normal aging. | Reported limitations included a small sample size, potential self-selection bias, underdiagnosis of long COVID-19, limited technological familiarity among older adults, and lack of access to internet-enabled devices among some patients. |
| Victoria et al. (2025) [5] | United States. | Randomized controlled trial. | 110 participants with long COVID-19 enrolled  98 participants completed at least one study visit (49 intervention, 49 control). | AKL-T01 (EndeavorOTC®) – a videogame-based digital therapeutic designed to enhance attention and executive control. | Cognitive symptoms, including attention deficits, processing speed, and executive function (task-switching).  Psychological symptoms, including anxiety and fatigue. Health-related quality of life. | No significant improvement in sustained attention.  Significant improvements in processing speed, task-switching ability,  functional disability,  health-related quality of life, fatigue (especially cognitive fatigue), and anxiety. | Reported limitations included a heterogeneous sample, absence of an active control group, a short follow-up period, and uncertainty regarding the long-term sustainability of the observed benefits. |
| Cano et al. (2024) [6] | Barcelona, Spain. | Pilot study of a non-randomized controlled trial. | 31 adults with PCC. | A multimodal IVR-based intervention including cognitive training, physical exercise, and mindfulness-based intervention (MBI). | Cognitive symptoms included disturbances in global cognition, attention, processing speed, memory, and executive function.  Psychological symptoms included anxiety, depression, and fatigue. | Experimental group showed significant improvements in global cognition, processing speed, episodic memory, attention, and executive function.  Significant reductions in depression symptoms (PHQ-9) in the experimental group but no changes in the control group​. | Reported limitations included the non-randomized design, small sample size, and lack of control for external factors such as medication use and prior experience with technology. |
| Schröder et al. (2023) [7] | Germany. | Observational cross-sectional study. | 342 patients with long COVID-19. | Digital tools in general for addressing symptoms related to Long COVID, including telehealth, mobile apps, and virtual reality. | Acceptance of eHealth interventions for symptom management and its predictors; depression, anxiety, fatigue, memory disturbances and concentration difficulties; and other symptoms: breathlessness, cough, headache, pain in limbs, tachycardia, dizziness, and others. | High acceptance of digital interventions, especially among patients with mental illness.  Key predictors of acceptance include trust in digital tool and perceived usefulness.  Female participants and those with prior experience using digital tools were more likely to express intention to use. | Reported limitations included the cross-sectional design limiting causal inferences, potential bias from self-reported data, limited generalizability due to a predominantly female sample, and heterogeneous access to the internet and digital tools. |
| Stavrou et al. (2023) [8] | Larissa, Greece. | Randomized controlled trial. | 20 patients after hospitalization due to COVID-19. | VR with cognitive training combined with physical exercise. | Global cognition; Patterns of sleep; Anthropometric and functional measures; Limb fatigue; Dyspnea; and Vital signs. | Patients from both arms performed equally on the VR cognitive exercises.  VR combined with exercised showed improvements in breathlessness.  No effect on cognitive measurements was reported.  No differences were seen on limb fatigue level. | Reported limitations included no improvement in cognitive function, which may indicate either a limitation in the VR training or mild cognitive impairment, all participants being infected with the less neurotropic COVID-19 delta variant, a small sample size, a short follow-up period, and the possibility that sleep disturbances and cognitive impairment influenced the results. |
| Ennis et al. (2023) [9] | United Kingdom. | Protocol for a randomized controlled trial. | Not mentioned  Targets long COVID-19 patients. | REGAIN online exercise and behavioural support intervention. | Cognitive symptoms: Brain fog, reduced concentration, memory disturbances.  Psychological symptoms: anxiety, depression, sleep disturbances, fatigue, fear of relapse, post-exertional malaise (PEM).  Quality of life and physical function. | As this is a randomized clinical trial protocol, no results are available yet. | Reported limitations included heterogeneity among patients, scarce literature, challenges with technological literacy, safety concerns regarding unsupervised exercise sessions, difficulties maintaining engagement throughout the study, and potential patient dropout due to fatigue. |
| Rinn et al. (2023) [10] | Germany. | Scoping review. | Patients with Long-COVID or post-COVID symptoms. | Digital health tools, including telerehabilitation programs, online peer-support groups, and other remote interventions. | Psychological symptoms: Anxiety, depression, post-traumatic stress, fatigue, loneliness, and emotional well-being.  Quality of life and functional status.  Pulmonary function and breathlessness. | Digital interventions improved fatigue, breathlessness, physical activity, health perception, and trust in health professionals, with mixed results for anxiety and depression. No improvements were noted in bowel, bladder, concentration, short-term memory issues, or unpleasant dreams, and long-term improvements were limited, with only a small percentage of patients achieving full health. | Reported limitations included small sample sizes, lack of controlled studies, heterogeneity in symptoms and interventions, limited patient safety measures, uncertainty about online peer support reliability, and potential omissions of important studies due to missing search terms. |
| León-Herrera et al. (2024) [11] | Zaragoza, Spain. | Randomized controlled trial. | 134 patients with long COVID-19. | Tele-rehabilitation program with weekly group videoconferences and an online platform (Moodle) for additional resources. | Cognitive symptoms: impaired global cognitive function, brain fog, memory disturbances, executive function problems.  Psychological symptoms: anxiety, depression, and sleep disturbances  Quality of life and physical function.  Personal constructs: Self-efficacy, health literacy and patient activation. | Significant improvement in mental health-related quality of life.  Increased self-efficacy was associated with greater improvement in mental health-related quality of life.  Cognitive function, emotional well-being, and physical health were noted but not statistically significant. | Reported limitations included variable adherence to the program, lack of participant blinding, potential confounding variables such as differences in baseline symptoms and functional status, and a majority of female participants. |
| Lai et al. (2024) [12] | Taiwan. | Randomized controlled trial. | 182 Long COVID patients. | A telerehabilitation training program to enhance physical activity using a mobile phone application and weekly remote reminders. | Psychological symptoms related to positive feelings, thinking, learning, self-esteem, bodily image and appearance, negative feelings, and spirituality.  Cognitive symptoms such as memory, concentration. | Improvements in physical activity amounts, exercise self-efficacy, and sleep quality.  No relevant differences between other measurements. | Reported limitations included a short-term study design, lack of pre- and post-intervention COVID-19 symptom assessment, reduced adherence, reliance on self-reported data, baseline differences between groups, and results likely limited to younger populations without multimorbidity or severe COVID-19. |
| Hatcher et al. (2022) [13] | Ottawa, Canada. | Protocol for a randomized controlled trial. | 152 patients with long COVID-19. | Electronic case management (ECM) platform. | Cognitive symptoms: Executive function, attention, memory, and verbal fluency  Psychological symptoms: anxiety, depression, sleep disturbances, PTSD, and fatigue.  Other symptoms: pain and breathlessness  Additionally, well-being, quality of life, and alcohol/substance use were considered. | As this is a randomized clinical trial protocol, no results are available yet. | Reported limitations included scarce literature on long COVID-19 treatment options, concerns about privacy and confidentiality, and challenges related to virtual visits. |
| Müllenmeister et al. (2024) [14] | Lower Saxony, Germany. | Pilot study of a randomized controlled trial. | 120-150 patients with long COVID-19. | Digital intervention using teletherapy and prerecorded videos for Occupational Therapy (OT). | Feasibility and acceptability of the study.  Cognitive symptoms: Brain fog, fatigue, memory, concentration, attention.  Quality of life, social participation, occupational performance, and satisfaction. | Results unavailable. Study is ongoing. | Pilot study to demonstrate feasibility and acceptability  Small sample size that limits generalizability and study power  Non-blinded design |
| Daniels et al. (2024) [15] | The included studies came from various countries, notably mentioning Spain, China, and the USA. | Scoping review | The review encompasses patients who have suffered from COVID-19, particularly those experiencing persistent symptoms following the illness. | The digital interventions varied but primarily focused on mHealth (mobile health apps), telerehabilitation, virtual rehabilitation, online platforms, and other digital tools. The duration and frequency of interventions varied among studies. Typically, interventions included sessions occurring several times a week over multiple weeks, although specific details were not standardized across all studies | Psychological symptoms included anxiety, sleep quality, depression, symptom burden, fear, fatigue  Cognitive functioning  Functional outcomes included strength assessment, cardiopulmonary fitness, balance, physical activity level, level of independence  Quality of life  Social support  Feasibility, usability and satisfaction with digital tools | Included studies revealed improvements in several key outcomes, including physical function enhancement; reduced anxiety, depression, and insomnia; improved sleep quality; increased cognitive function; reduced dyspnea, improved muscle strength, better cardiopulmonary fitness; improved quality of life | Heterogeneity among included studies  Observational studies were not included  Many interventions restricted to experimental settings rather than clinical rehabilitation services  Lack of familiarity with technology among patients  Limitations related to financial constraints  Impact of sex and gender on acceptability and efficacy of the interventions was not explored |
| Tsang & Tabio (2024) [16] | New York, United States | Pilot study of an observational prospective study | 11 patients with long COVID-19 (2 dropouts) | Telehealth group sessions providing peer social support for adults with long COVID, verified COVID-19 information and recovery strategies, and evidence-based cognitive behavioral treatment (CBT) | Qualitative assessment of recipient satisfaction  Psychological distress  Fatigue  Attrition and attendance rates | As this is a randomized clinical trial protocol, no results are available yet. | Reported limitations included a small sample size, lack of a control group, and a design that did not allow for the evaluation of treatment efficacy. |
| Groenveld et al. (2022) [17] | Nijmegen, Netherlands. | Observational prospective study. | 48 patients with long COVID-19. | Virtual reality physical exercise and virtual reality mental exercises. | Feasibility, including acceptability, usability, tolerability, and safety of VR exercises.  Psychological symptoms: Anxiety, depression, stress, feelings regarding health.  Cognitive symptoms: perception of cognitive function, memory, attention.  Quality of life, daily functioning, participation, and satisfaction. | One patient did not start VR, and seven withdrew, mostly due to dizziness. Most patients reported positive contributions of VR to recovery, with 67% global satisfaction. Physical exercise frequency decreased over time, while cognitive and relaxation exercises remained stable. Physical performance and quality of life improved, but there was no change in cognitive measures. 70% reported VR-related adverse effects, including dizziness (45%) and headaches (21%). | Reported limitations included scarce literature on long COVID rehabilitation at the time of the study, the intense support from the research team being unfeasible for most rehabilitation services, lack of a multiprofessional approach, limited patient participation due to unfamiliarity with VR, technical challenges affecting the rehabilitation experience, and the absence of a control group, limiting conclusions on treatment effectiveness. |
| Smith et al. (2023) [18] | United Kingdom. | Observational prospective study. | 601 patients with long COVID-19. | Blended rehabilitation program including a web-based rehabilitation service with on-demand exercise videos, weekly livestreamed group exercise classes, educational webinars and resources for symptom management, and in-person community-based rehabilitation sessions. | Psychological symptoms: Anxiety, depression, emotional distress, fatigue, mental well-being.  Cognitive symptoms: Brain fog, concentration issues  Breathlessness, functional capacity, quality of life and healthcare utilization metrics. | Key findings of the study included significant improvements in all measured outcomes, with reduced breathlessness, better health-related quality of life, decreased healthcare utilization, and improvements in physical function. | Reported limitations included the absence of a control group, limited diversity, short follow-up period, exclusion of specific populations (e.g., chronic fatigue syndrome), unbalanced heterogeneity in the severity of COVID-19 infection, and no stratification by disease severity. |
| Al-Jabr et al. (2022) [19] | Suffolk, United Kingdom. | Protocol for a randomized controlled trial. | 60 participants with long COVID-19. | Online therapy intervention involving videoconferences and telephone sessions, which can be 1:1 or group sessions. | Psychological symptoms: Anxiety, depression, fatigue, self-efficacy.  Quality of life. | As this is a randomized clinical trial protocol, no results are available yet. | Reported limitations included the lack of blinding for participants or researchers, a small sample size, and scarce literature on long COVID-19. |
| Prudent et al. (2024) [20] | NA | Narrative review. | Long COVID syndrome. | Virtual Reality (VR); Cognitive Behavioral Therapy (CBT); and Trauma-focused digital therapies (TF-CBT). | Anxiety, depression, and post-traumatic stress disorder (PTSD). | Significant improvements in psychological symptoms resulting from the digital interventions among included studies, especially in patients with PTSD. | Reported limitations included a lack of quantitative data supporting study claims, particularly regarding PTSD, limited generalizability due to studies focusing on specific populations (e.g., pediatric cohorts or refugees), and scarce literature on long-term digital therapies for long COVID patients. |
| Montes-Ibarra et al. (2024) [21] | Alberta, Canada. | Protocol for a pilot randomized controlled trial. | 40 patients with long COVID-19. | Web-based wellness platform called My Viva Plan (MVP)® targeting nutrition, physical activity, and mindfulness using CBT techniques. | The study assessed various outcomes, including feasibility, quality of life, mental health (anxiety, depression), physical health (body composition, physical functioning), nutritional status, PCC symptom burden, and self-efficacy. | As this is a randomized clinical trial protocol, no results are available yet. | Reported limitations included dietary assessment based on a single 24-hour recall, lack of monitoring the time spent in the app, financial limitations restricting MRI use in the intervention group, and engagement tracked only by users completing daily reflections. |
| Krotz et al. (2023) [22] | Germany. | Protocol for a randomized controlled trial. | 600 patients with long COVID-19. | Online learning platform with three modalities: patient information only; patient information plus a digital workbook including practical exercises; and all previous ones plus a once-weekly online seminars and discussion groups. | The study assessed various outcomes, including sick leaves, quality of life, psychological symptoms (depression, anxiety, burnout, sleep quality, well-being), social support, physical fitness, work ability, and breathlessness. | As this is a randomized clinical trial protocol, no results are available yet. | Reported limitations included potential dropouts due to lack of human support in the low-intensity group, risk of unblinding if participants communicated with each other, heterogeneity of the patient sample, and challenges in recruitment. |
| Eilam-Stock et al. (2021) [23] | New York, United States. | Case series study. | 2 patients with long COVID-19. | Remotely supervised tDCS (transcranial direct current stimulation). | The study assessed various outcomes, including fatigue, brain fog, pain, anxiety, and depression. | Both patients showed significant improvements in cognitive functioning, depression, and fatigue; one reported increased anxiety, while the other reported improvements in emotional and functional domains. | Reported limitations included the intervention being feasible but on a small scale, a small sample size limiting conclusions on treatment effectiveness, lack of a control group, and no long-term follow-up. |
| Vanova et al. (2024) [24] | United Kingdom. | Protocol for a Randomized Controlled Trial. | 120 patients with persistent cognitive impairment due to long COVID-19. | Telehealth-delivered cognitive rehabilitation (COVID-Rehab). | Cognitive symptoms: Executive function, attention, processing speed, memory.  Psychological symptoms: Anxiety, depression, fatigue, sleep quality, post-exertional malaise. | As this is a randomized clinical trial protocol, no results are available yet. | Reported limitations included non-blinded participants, the study being limited to patients aged 30 to 60 years, and potential attrition. |
| Pepa et al. (2022) [25] | Italy. | Observational prospective study. | The platform had an unknown number of unique visitors, with 220 visits and 623 page views per day. Fifty participants completed a user satisfaction survey, and the platform targets patients recovering from COVID-19. | Web-based tele-health platform for therapeutic exercise and education. | The study assessed various outcomes, including fatigue, anxiety, user satisfaction, self-reported improvement, page views per day, and protocol completion rate. | 80% of participants reported fatigue during daily activities, with a perceived improvement of 6.5/10. 64% had moderate to severe anxiety, and 54% reported improvement post-training. There were no side effects, and user satisfaction was high (4.4/5), though some reported audio issues on the platform. | Reported limitations included the absence of a control group, the possibility that self-reported improvements may not align with objective clinical assessments, limited follow-up, the need for in-person support for patients with severe disease, and concerns regarding privacy and cybersecurity. |

PACS: post-acute COVID-19 syndrome; NA: not applicable; PROMs: Patient-Reported Outcome Measures; VR: virtual reality; IMGV: Virtual Integrative Medical Group Visits; PCC: Post-COVID-19 Condition; IVR: immersive virtual reality; MBI: mindfulness-based intervention; REGAIN: Rehabilitation Exercise and psycholoGical support After covid-19 InfectioN; PEM: post-exertional malaise; ECM: electronic case management; OT: occupational therapy; CBT: cognitive behavioral treatment/therapy; TF-CBT: trauma-focused digital therapies; PTSD: post-traumatic stress disorder; MRI: magnetic resonance imaging.

**References**

1. Dahmen A, Keller FM, Derksen C, Rinn R, Becker P, Lippke S. Screening and assessment for post-acute COVID-19 syndrome (PACS), guidance by personal pilots and support with individual digital trainings within intersectoral care: a study protocol of a randomized controlled trial. BMC Infect Dis 2022;22(1):693. doi: 10.1186/s12879-022-07584-z

2. Murray E, Goodfellow H, Bindman J, Blandford A, Bradbury K, Chaudhry T, Fernandez-Reyes D, Gomes M, Hamilton FL, Heightman M, Henley W, Hurst JR, Hylton H, Linke S, Pfeffer P, Ricketts W, Robson C, Singh R, Stevenson FA, Walker S, Waywell J. Development, deployment and evaluation of digitally enabled, remote, supported rehabilitation for people with long COVID-19 (Living With COVID-19 Recovery): protocol for a mixed-methods study. BMJ Open 2022 Feb 1;12(2):e057408. doi: 10.1136/bmjopen-2021-057408

3. Widmann CN, Henkel C, Seibert S. “Brain Fog” After COVID-19 Infection: How the Field of Neuropsychology Can Help Clear the Air. In: Rezaei N, editor. The COVID-19 Aftermath: Volume II: Lessons Learned Cham: Springer Nature Switzerland; 2024. p. 59–76. doi: 10.1007/978-3-031-61943-4_5ISBN:978-3-031-61943-4

4. Mata R, Okanlawon Bankole A, Barnhill J, Roth I. A descriptive exploration of younger and older adults’ experiences of Integrative Medical Group Visits for Long COVID. Aging Health Res 2023;3(2):100137. doi: https://doi.org/10.1016/j.ahr.2023.100137

5. Victoria LW, Oberlin LE, Ilieva IP, Jaywant A, Kanellopoulos D, Mercaldi C, Stamatis CA, Farlow DN, Kollins SH, Tisor O, Joshi S, Doreste-Mendez R, Perlis RH, Gunning FM. A digital intervention for cognitive deficits following COVID-19: a randomized clinical trial. Neuropsychopharmacology 2025;50(2):472–479. doi: 10.1038/s41386-024-01995-z

6. Cano N, Gómez-Hernández J, Ariza M, Mora T, Roche D, Porras-Garcia B, Garolera M. A multimodal group-based immersive virtual reality intervention for improving cognition and mental health in patients with post-covid-19 condition. A quasi-experimental design study. Front Psychol 2024;15. Available from: https://www.frontiersin.org/journals/psychology/articles/10.3389/fpsyg.2024.1441018

7. Schröder J, Bäuerle A, Jahre LM, Skoda E-M, Stettner M, Kleinschnitz C, Teufel M, Dinse H. Acceptance, drivers, and barriers to use eHealth interventions in patients with post-COVID-19 syndrome for management of post-COVID-19 symptoms: a cross-sectional study. Ther Adv Neurol Disord England; 2023 May;16:17562864231175730.

8. Stavrou VT, Vavougios GD, Kalogiannis P, Tachoulas K, Touloudi E, Astara K, Mysiris DS, Tsirimona G, Papayianni E, Boutlas S, Hassandra M, Daniil Z, Theodorakis Y, Gourgoulianis KI. Breathlessness and exercise with virtual reality system in long-post-coronavirus disease 2019 patients. Front Public Health 2023;11. Available from: https://www.frontiersin.org/journals/public-health/articles/10.3389/fpubh.2023.1115393

9. Ennis S, Heine P, Sandhu H, Sheehan B, Yeung J, McWilliams D, Jones Christina and Abraham C, Underwood M, Bruce J, Seers K, McGregor G. Development of an online intervention for the Rehabilitation Exercise and psycholoGical support After covid-19 InfectioN (REGAIN) trial. NIHR Open Res England; 2023 Jul;3:10.

10. Rinn R, Gao L, Schoeneich S, Dahmen A, Anand Kumar V, Becker P, Lippke S. Digital Interventions for Treating Post-COVID or Long-COVID Symptoms: Scoping Review. J Med Internet Res 2023 Apr 17;25:e45711. doi: 10.2196/45711

11. León-Herrera S, Oliván-Blázquez Bárbara and Sánchez-Recio R, Méndez-López F, Magallón-Botaya R, Sánchez-Arizcuren R. Effectiveness of an online multimodal rehabilitation program in long COVID patients: a randomized clinical trial. Arch Public Health England; 2024 Sep;82(1):159.

12. Lai C-Y, Lin C-H, Chao T-C, Lin C-H, Chang C-C, Huang C-Y, Chiang S-L. Effectiveness of a 12-week telerehabilitation training in people with long COVID: A randomized controlled trial. Ann Phys Rehabil Med 2024;67(5):101853. doi: https://doi.org/10.1016/j.rehab.2024.101853

13. Hatcher S, Werier J, Edgar NE, Booth J, Cameron DWJ, Corrales-Medina V, Corsi D, Cowan J, Giguère P, Kaluzienski M, Marshall S, Mestre T, Mulligan B, Orpana H, Pontefract A, Stafford D, Thavorn K, Trudel G. Enhancing COVID Rehabilitation with Technology (ECORT): protocol for an open-label, single-site randomized controlled trial evaluating the effectiveness of electronic case management for individuals with persistent COVID-19 symptoms. Trials 2022;23(1):728. doi: 10.1186/s13063-022-06578-1

14. Müllenmeister C, Stoelting A, Schröder D, Schmachtenberg T, Ritter S, El-Sayed I, Steffens S, Klawonn F, Klawitter S, Homann S, Mikuteit M, Berg C, Behrens G, Hummers E, Cook A, Müller F, Dopfer-Jablonka A, Happle C. Evaluating the Feasibility, Acceptance, and Beneficial Effects of Online Occupational Therapy for Post–COVID-19 Condition: Protocol for a Randomized Controlled Trial (ErgoLoCo Study). JMIR Res Protoc 2024;13:e50230. doi: 10.2196/50230

15. Daniels K, Mourad J, Bonnechère B. Exploring the Use of Mobile Health for the Rehabilitation of Long COVID Patients: A Scoping Review. Healthcare 2024 Feb 10;12(4):451. doi: 10.3390/healthcare12040451

16. Tsang W, Tabio L. Feasibility of a Manualized Long COVID Telehealth Group Therapy within a Metropolitan Outpatient Rehabilitation Psychology Service. Arch Phys Med Rehabil 2024;105(4):e141. doi: https://doi.org/10.1016/j.apmr.2024.02.400

17. Groenveld T, Achttien R, Smits M, de Vries M, van Heerde R, Staal B, van Goor H. Feasibility of Virtual Reality Exercises at Home for Post–COVID-19 Condition: Cohort Study. JMIR Rehabil Assist Technol 2022;9(3):e36836. doi: 10.2196/36836

18. Smith JL, Deighton K, Innes AQ, Holl M, Mould L, Liao Z, Doherty P, Whyte G, King JA, Deniszczyc D, Kelly BM. Improved clinical outcomes in response to a 12-week blended digital and community-based long-COVID-19 rehabilitation programme. Front Med (Lausanne) 2023;10. Available from: https://www.frontiersin.org/journals/medicine/articles/10.3389/fmed.2023.1149922

19. Al-Jabr H, Windle K, Thompson DR, Jenkins ZM, Castle DJ, Ski CF. Long COVID Optimal Health Program (LC-OHP) to Enhance Psychological and Physical Health: Protocol for a Feasibility Randomized Controlled Trial. JMIR Res Protoc 2022;11(5):e36673. doi: 10.2196/36673

20. Prudent C, Batt M, Gamraoui S, Danan J-L, Klos J-Y. Télépsychothérapie et COVID-long, une indication envisageable ? Annales Médico-psychologiques, revue psychiatrique 2024;182(6):520–530. doi: https://doi.org/10.1016/j.amp.2023.11.012

21. Montes-Ibarra M, Godziuk K, Thompson RB, Chan CB, Pituskin E, Gross DP, Lam G, Schlögl M, Felipe Mota J, Ian Paterson D, Prado CM. Protocol for a pilot study: Feasibility of a web-based platform to improve nutrition, mindfulness, and physical function in people living with Post COVID-19 condition (BLEND). Methods 2024;231:186–194. doi: https://doi.org/10.1016/j.ymeth.2024.10.004

22. Krotz A, Sosnowsky-Waschek N, Bechtel S, Neumann C, Lohkamp M, Kovacs G, Genser B, Fischer JE. Reducing sick leave, improving work ability, and quality of life in patients with mild to moderate Long COVID through psychosocial, physiotherapeutic, and nutritive supportive digital intervention (MiLoCoDaS): study protocol for a randomized controlled trial. Trials 2023;24(1):798. doi: 10.1186/s13063-023-07819-7

23. Eilam-Stock T, George A, Lustberg M, Wolintz R, Krupp LB, Charvet LE. Telehealth transcranial direct current stimulation for recovery from Post-Acute Sequelae of SARS-CoV-2 (PASC). Brain Stimul 2021;14(6):1520–1522. doi: https://doi.org/10.1016/j.brs.2021.10.381

24. Vanova M, Patel AMR, Scott I, Gilpin G, Manning EN, Ash C, Wittenberg P, Lim J, Hoare Z, Evans R, Bray N, Kipps CM, Devine C, Ahmed S, Dunne R, Koniotes A, Warren C, Chan D, Suarez-Gonzalez A. Telehealth-delivered cognitive rehabilitation for people with cognitive impairment as part of the post-COVID syndrome: protocol for a randomised controlled trial as part of the CICERO (Cognitive Impairment in Long COVID: Phenotyping and Rehabilitation) study. Trials 2024;25(1):704. doi: 10.1186/s13063-024-08554-3

25. Pepa L, Pigliapoco M, Bisoglio P, Lambertucci A, Coccia M, Ercolani L, Aringolo M, Hibel M, Gastaldi A, Andrenelli E, Cima R, Spalazzi L, Ceravolo MG, Capecci M. Therapeutic Exercise Protocols for People Recovering After Covid-19: A Tele-Health Approach. In: Bettelli A, Monteriù A, Gamberini L, editors. Ambient Assisted Living Cham: Springer International Publishing; 2022. p. 355–362.
